# Assessment of current virotherapeutic application schemes: “hit hard and early” versus “killing softly”?

**DOI:** 10.1038/mto.2015.18

**Published:** 2015-11-04

**Authors:** Benjamin Ruf, Ulrich M Lauer

**Affiliations:** 1 Department of Internal Medicine I, University Hospital Tuebingen, Tuebingen, Germany

## Abstract

Over the past two decades, a considerable amount of oncolytic vector families has entered numerous clinical trials. However, to this date, the field has not yet been able to come to a common understanding regarding the best possible ways to administer oncolytic viruses to cancer patients. This is mainly due to the fact that so far clinical trials being designed for head-to-head comparisons (such as using two different virotherapeutics originating from two distinct virus families being applied via identical routes in the same types of cancer) are still missing. Hence, there is no consent (i) on the best route of virotherapeutics administration (*e.g.*, systemic versus intratumoral), (ii) on the virus dosages to be applied, (iii) on dosing intervals, and (iv) on the numbers of repetitive courses of virus administration. As the detailed comparison of clinical virotherapy trial regimens is time-consuming and complex, we here present an overview of current state-of-the-art virotherapeutic application schemes. Notably, our comprehensive assessment culminates in raising two rough classifications of virotherapeutic strategies, *i.e.*, “hit hard and early” versus “killing softly”. In order to find out which one of these two gross alternatives might be most successful for each and every tumor entity, we here suggest the implementation of phase 1/2 studies, which primarily aim at a repetitive sampling and analysis of tumor samples in cancer patients treated with oncolytic viruses reading out (i) virus-specific, (ii) tumor-specific as well as (iii) immunotherapeutic parameters. On this basis, a rational design of significantly improved virotherapeutic application schemes should be possible in the future.

## Introduction

The field of oncolytic virotherapy undoubtedly has made formidable progress since first ever replication-competent, genetically engineered viruses have entered preclinical and clinical testing in the 1990s.^1^ The first clinical trials with such modified/attenuated virus pathogens used as oncolytic vectors primarily had to address numerous safety concerns. But ever since, oncolytic viruses have proven to constitute generally well-tolerated novel biological anticancer agents.^2^

However, when coming to the efficiency of the oncolytic paradigm many limitations of those first-generation virotherapeutics regarding antitumor efficacy became obvious.^3^ Accordingly, next-generation oncolytics were designed (i) to enhance tumor specificity, (ii) to express efficiency-boosting transgenes, such as suicide genes or immunomodulatory cytokines, or (iii) to coat viruses as camouflage (to avoid rapid neutralization when getting in contact with the highly effective antiviral host immune response).^4^ Latest evidence suggests that antitumor activity of oncolytic viruses is not solely dependent on pathogen-mediated direct/specific infection and (onco-)lysis of malignant cells but is also capable of triggering an adaptive antitumor immune response. In this context, current evidence suggests that the mechanisms of action of virotherapeutics can be attributed at least partly (i) to a profound exposure of antigenic tumor epitopes being released in huge amounts throughout the oncolytic process, (ii) to a subsequent inflammatory tumor infiltration as well as (iii) to the induction of a T-cell-mediated antitumor immune response (see original work^5–8^ as well as data reviewed in ref. 9).

Current knowledge gained (i) from recent clinical virotherapy trials^1,10^ as well as (ii) from the role of oncolytic viruses in establishing a long-lasting, highly effective antitumor immune response^3,11^ have been reviewed already. However, a comprehensive evaluation of current application schemes is still missing. Accordingly, every sponsor/investigator assigned with the sophisticated task to design a “fresh” protocol for a new clinical virotherapy trial still has to address the same, hitherto quite challenging questions: (i) by which route should I administer my respective study virus?, (ii) which dosages should be applied?, (iii) what are the optimal dosing intervals?, (iv) for how long should reapplications take place? Thus, the design of virotherapy studies is still highly complex and full of pitfalls. Failure in study success simply can be due to suboptimal or even “wrong” application schemes.

How to handle this situation? There are two approaches which might be instrumental in the establishment of future “gold standard” virotherapy application regimens: First, a comprehensive assessment of current application schemes could help to identify basic application approaches and assign these to successful regimens. In this line, we here identified two main paradigms of current virotherapeutic treatment approaches: (i) on the one hand, the single-shot application paradigm paraphrased as “hit hard and early”; (ii) on the other hand a multiple/repetitive applications of virotherapeutics euphemistically referred to as a “soft killing” paradigm. Going beyond such a systematic analysis of the current trial situation, we also discuss the implementation of further phase 1/2 studies which primarily should aim at a repetitive sampling and analysis of tumor samples in cancer patients treated with oncolytic viruses. This more “vigorous” virotherapeutic trial approach might pave the way for a better understanding of the biological effects of each and every study virus in the context of its respective application scheme. Such a comprehensive strategy might then enable a step-by-step improvement/optimization of current application regimens.

## Methods

We analyzed clinical trials using oncolytic viruses as a single-agent monotherapy, excluding studies combining viruses with other modalities, such as chemotherapy, radiation, or immunomodulating agents. For our investigation, we used the http://clinicaltrials.gov/ service of the US National Institutes of Health. Thus, only protocols which are available on this website are cited within this review. Furthermore, we exclusively analyzed trials that provided a substantial description regarding the respective application schemes (*i.e.*, route of virus administration, dosage, dosing intervals, and numbers of repetitive courses of virus dosages).

Facing the plethora of virotherapy trials, we then had to carefully select those trials with (probably) interesting application schemes, balancing between trying to avoid unnecessary redundancy and nevertheless providing a comprehensive review for the reader. We not only included completed, but also still ongoing studies. Preferentially, we emphasized on studies which have already been published or have been presented at major meetings (*e.g.*, at ASCO Annual Meetings). We apologize to researching investigators whose work could not been mentioned.

Following the compilation of all relevant single-agent virotherapy trials, we focused on the five most prominent virotherapeutic vector families (*Measles/Paramyxoviridae, Vaccinia/Poxviridae, Herpesviridae, Adenoviridae*, *and Reoviridae*), which we reviewed below in a descriptive manner and which are also displayed in easy-to-read figures. All other trials encompassing vector systems in their very early clinical development as well as unpublished trials are further displayed in the same style in supplementary figures but not presented in text (due to space restrictions).

Table 1 presents a quick overview of the application schemes of all trials covered by our assessment (sorted by virus families). In addition, numerous application schemes are displayed graphically in the figure section (sorted by oncolytic vector platforms as well as by the respective identifiers provided by http://clinicaltrials.gov/).

## Findings

### Measles vaccine viruses (MV)-based oncolytic monotherapy

To date, two Edmonston strain-derived measles vaccine viruses (MV-Edm) are intensively explored clinically, namely MV-CEA and MV-NIS. MV-CEA encodes for carcinoembryonic antigen (CEA) which can be employed as a marker gene for viral gene expression *in vivo*.^12^ MV-NIS encodes for the human thyroidal sodium iodide symporter and can be used both for noninvasive imaging for viral gene expression, *e.g.*, by SPECT/CT and for radiovirotherapy, *e.g.*, with ionizing gamma-radiation-emitter ^131^I radioiodine.^13^

#### Repetitive application schemes for MV.

**Figure 1:**
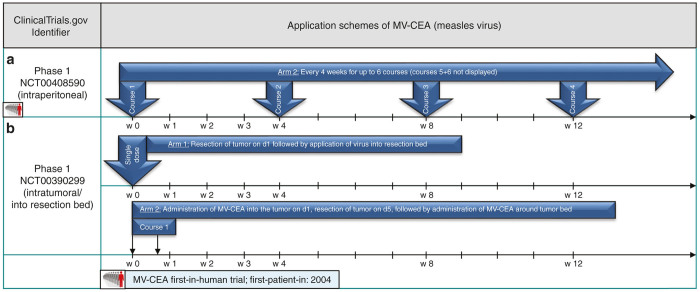
Selected application schemes of MV-CEA (**a**+**b**): (**b**) published by Galanis *et al*.^14^

First results of a MV-CEA dose-escalating clinical trial on refractory ovarian cancer (NCT00408590) were published in 2010.^14^ Patients were treated with MV-CEA through an intraperitoneal (i.p.) catheter every 4 weeks for up to six courses (application scheme depicted in Figure 1a). Treatment was well tolerated, as no dose-limiting toxicities occurred. Antitumor activity led to stable disease in 14/21 patients with a median duration of 92.5 days. CEA marker-gene detection was reported in peritoneal fluid and serum favorably in patients receiving high dosages of the study virus.

**Figure 2:**
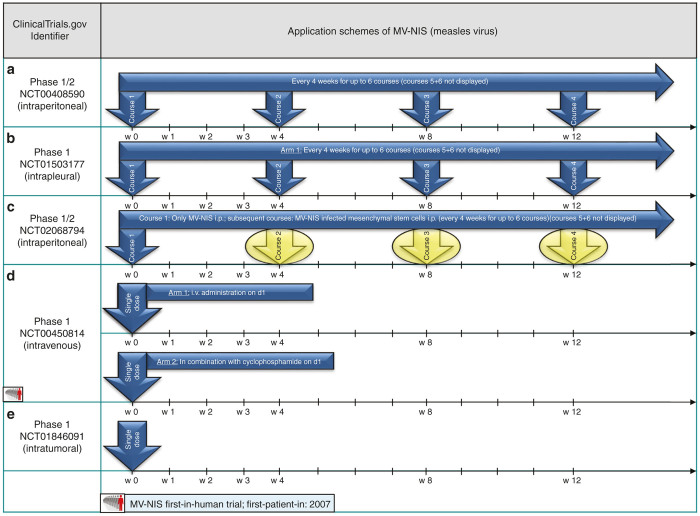
Selected application schemes of MV-NIS (**a**–**e**): (**a**) published by Galanis *et al*.^15^ (**d**) published by Russell *et al*.^17^

Encouraged by these results, also MV-NIS was applied to women diagnosed with drug-resistant ovarian cancer in a subsequent part of the same phase 1/2 trial (NCT00408590). Again, MV-NIS was administered into the peritoneal cavity every 4 weeks for up to six courses (see Figure 2a) and results regarding safety and efficacy correlated well with the previous MV-CEA trial but additional information was gained by radio imaging of viral gene expression.^15^

Pointing out a high level of protocol adherence, another phase 1 trial on malignant pleural mesothelioma (NCT01503177) also applied MV-NIS with six courses every 4 weeks into the pleural cavity (see Figure 2b).

Notably, also an innovative application scheme is included in the protocol of another MV-NIS clinical trial on therapy-resistant ovarian cancer (NCT02068794): intraperitoneal application on the first course with MV-NIS is followed by subsequent courses every 4 weeks for up to six courses with MV-NIS-infected mesenchymal stem cells. These cell-based virus delivery systems are administered intraperitoneally as well (see Figure 2c). The use of virus-loaded cell carriers to evade premature sequestration of virotherapeutics by the host immune response (*e.g.*, by preexisting antimeasles antibodies) after an initial uncoated loco-regional (*i.e.*, intraperitoneal) measles infection already has shown very promising results in a xenograft mouse model. Tumor-specific infiltration of parenchyma with subsequent virus delivery by measles virus-infected mesenchymal stem cells was found to prolong overall survival when compared to “naked” infectious virus particles. Therefore, a strong preclinical rationale had been built for exploring this innovative application design in the clinical setting.^16^

#### MV single-shot application schemes.

Russell *et al*.^17^ from the Mayo Clinic have recently presented a case report (NCT00450814) describing a durable complete remission of a patient with therapy-refractory multiple myeloma after a single shot of intravenous MV-NIS (see Figure 2d). MV-NIS expression allowed the investigators to monitor infection of disseminated tumor sites with subsequent vanishing of all detectable tumor masses. Another application of a single shot of MV-NIS in the treatment of head and neck cancer is part of a phase 1 trial (NCT01846091) but here administered by intratumoral injection (see Figure 2e). A trial with MV-CEA on brain and central nervous system tumors (NCT00390299) is using an altered application scheme. A single-shot application into the resection cavity after brain surgery is compared to a double-hit course with one application presurgery (via catheter) and another postsurgical intervention into the resection cavity (application scheme also depicted in Figure 1b).

As outlined above, insights gained from the MV-NIS trial on multiple myeloma serve as a prime example to substantiate the single-shot “hit hard and early” paradigm. Here, Russell and colleagues provided a proof-of-principle that a single shot of systemically administered MV at the maximum achievable dosage could lead to a complete clinical response even at advanced stages of disease. Key factors for a successful implementation of the single-shot paradigm were proposed to be the high dosage of infectious particles used for patient treatment (= “hit hard”) and no detectable amount of preexisting antivirus serum antibodies (= “hit early”; *i.e.*, prior to induction of a virus-specific immune response).

### Oncolytic poxviruses: vaccinia virus for tumor patients

State-of-the-art engineered oncolytic vaccinia viruses are derivatives of different vaccine strains (*e.g.*, Wyeth (JX-594), Western Reserve (JX-929), and Lister (GL-ONC1)) using different virus backbones for the design of tumor-selective, highly efficient (through arming with therapeutic transgenes) and safe oncolytic vectors.^18^

#### Repetitive application schemes for study virus JX-594.

**Figure 3:**
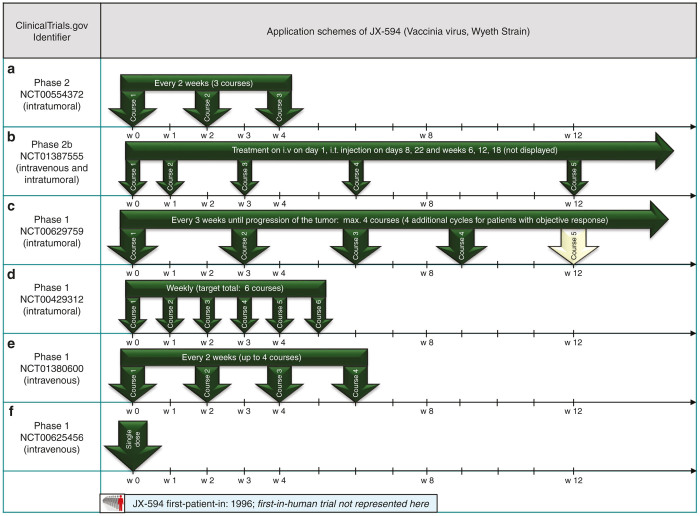
Selected application schemes of JX-594 (**a**-**f**): (**a**) published by Heo *et al*.,^68^ (**b**) presented at ASCO 2013,^20^ (**c**) published by Park *et al*.,^21^ (**d**) published by Hwang *et al*.,^7^ (**e**) presented at ASCO 2013,^22^ (**f**) published by Breitbach *et al*.^23^

JX-594 (pexastimogene devacirepvec, Pexa-Vec) is derived from the Wyeth vaccine strain and targeted to transformed (malignant) cells by deletion of viral thymidine kinase. Furthermore, JX-594 codes for the immunostimulatory cytokine gene hGM-CSF.^19^ Pexa-Vec is object of several phase 1/2 clinical trials, including a completed phase 2 trial on hepatocellular carcinoma (NCT00554372). In this trial, JX-594 was injected into the tumoral liver lesions for three courses, each 2 weeks apart (see Figure 3a). Treatment with JX-594 was generally well tolerated and overall survival was found to be dose-dependent, as the median overall survival was found to be 14.1 months for the high-dose receiving patients versus 6.7 months in the low-dose group. Of note, objective tumor responses were found in injected as well as noninjected tumor liver-lesions and JX-594 genomes could be detected in blood samples as late as 15–36 days after the initial injection, indicating ongoing in-patient virus replication. The application of JX-594 in this prime-boost setting is supported by the findings of an induced humoral antitumor immunity after virus infection, although tumor-specific cellular immune response was not assessed in this trial.^20^ Thus, further evaluation will be needed to attribute this application scheme as “killing softly” being defined as an ongoing tumor colonization and subsequent boosted antitumoral immune response.

Based on these results, a phase 2b randomized trial on heavily pretreated HCC patients (NCT01387555) was launched in 2011,^20^ using a more sophisticated application scheme: patients receive intravenous (i.v.) infusion of a high dosage of Pexa-Vec on day 1, followed by intratumoral application on d8 and d22, as well as 6, 12, and 18 weeks after initial treatment (see Figure 3b).

The corresponding phase 1 trial on primary and metastatic liver cancer (NCT00629759) used a dose-escalating design with intralesional injections of JX-594 every 3 weeks for a total of four injections (including extensions to a maximum of eight applications if objective responses occurred) (see Figure 3c). Park *et al.*^21^ reported the experience of dose-limiting toxicities at the highest dose level, but found JX-594 also in noninjected tissue; 9 of 10 patients had objective response (according to RECIST criteria).

In a phase 1 trial on metastatic melanoma (NCT00429312), JX-594 was injected intratumorally on a weekly basis for a total of six courses (see Figure 3d). In this proof-of-concept trial, replication of vaccinia virus was detectable in tumor samples as well as blood tests and detection of granulocyte-macrophage colony-stimulating factor (GM-CSF) was used as a marker for viral gene expression.^7^

Intravenous application of Pexa-Vec is also part of a phase 1 trial on advanced colorectal carcinoma (NCT01380600, Figure 3e) with i.v. infusions every 2 weeks for up to four courses. Presentation at ASCO 2013 reported that clearance of JX-594 was not more rapid in subsequent i.v. infusions, despite induction of a humoral immune response.^22^

Further application schemes can be found in registered but yet unpublished trials on Pexa-Vec: NCT01394939 (Supplementary Figure S1a): i.v. administration of JX-594 weekly for five courses (additional three infusion boosts possible). NCT02017678 (Supplementary Figure S1b) and NCT01636284 (Supplementary Figure S1c): systemic delivery of five courses Pexa-Vec in weekly intervals with continuance of infusions every 3 weeks until (radiographically assessed) progression of disease. NCT01469611 (Supplementary Figure S1d): dose-escalating study with i.v. administration of Pexa-Vec every 2 weeks until dose-limiting toxicities are observed.

#### Single-shot application schemes for JX-594.

A phase 1 trial on refractory solid tumors (including melanoma, lung cancer, renal cancer, and squamous cell carcinoma of the head and neck) was launched recently (NCT00625456, Figure 3f). JX-594 was administered intravenously in a single-shot treatment regimen. Breitbach *et al*.^23^ reported the observation of JX-594 delivery to disseminated tumor sites and found replication of virus vector and transgene expression to be dose-related. This trial therefore supports further evaluation of JX-594 in the single-shot scenario, although this early stage phase 1 trial rather focused on virus pharmacokinetics and transgene expression than assessment of patient survival data. These issues make it hard to speculate on potential benefits of either single-shot or prime-boost application schemes in case of JX-594 and further data have to be acquired covering virus biodistribution as well as antitumor humoral and cellular immune responses.

#### Study virus JX-929 single-shot application schemes.

A Western Reserve strain vaccinia virus including deletions in both tyrosine kinase^24^ and vaccinia growth factor (VGF)-gene-loci with additional insertion of a suicide gene-transgene coding for bacterial cytosine deaminase (CD) has entered clinical practice as JX-929 (vvDD-CDSR).^25^ In a phase 1 dose-escalating trial on advanced solid tumors (NCT00574977), patients received intratumoral injection of JX-929 as single-shot application (application scheme depicted in Supplementary Figure S1e). Results of this trial were most recently published^26^ and regarding safety concerns, one treatment-related severe adverse event was documented (1/16 patients). The investigators report evidence for vvDD replication also in lesions that were not injected, but clinical benefit was found to be limited to histological samples. Although these results can be ragarded as proof-of-concept (infectious particles had reached disseminated tumor sites), clinical outcome did not correspond with findings of virus dissemination; this suggests an even more “rigorous” sampling of tumor tissues in the future from both injected and noninjected tumor lesions in order to obtain a comprehensive readout on (i) virus-specific, (ii) tumor-specific as well as (iii) immunotherapeutic parameters.

#### Repetitive application schemes for GL-ONC1.

**Figure 4:**
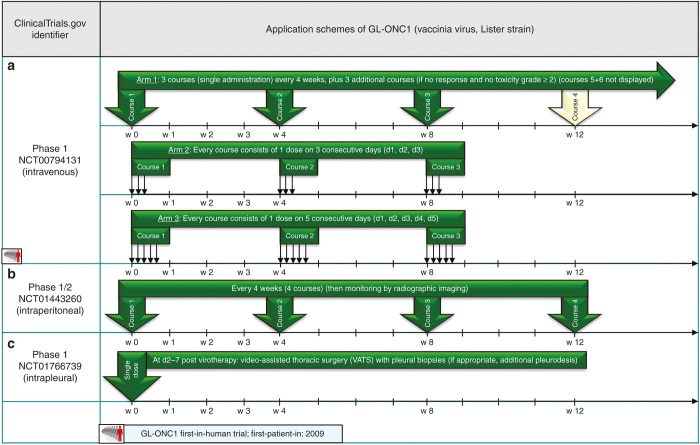
Selected application schemes for GL-ONC1 (**a**–**c**): (**a**) presented at ASCO 2013,^29^ (**b**) presented at ASCO 2013.^30^

GL-ONC1 is a Lister strain-derived Vaccinia virus encoding three marker genes (a fusion of Renilla luciferase-Aequorea green fluorescent protein, β-galactosidase, β-glucuronidase) that can be employed for diagnostic purposes,^27^ while deletion of thymidine kinase assures tumor selectivity. GL-ONC1 is currently undergoing clinical evaluation regarding safety and efficacy in several phase 1/2 trials using a variety of application schemes:

GL-ONC1 first in-human phase 1 trial in patients with advanced solid tumors (NCT00794131) has a dose-escalating study design subdivided into several cohorts of different application schemes (application schemes depicted in Figure 4a): application scheme no. 1 demands i.v. infusion of GL-ONC1 every 4 weeks for three courses (in case of no response and no toxicity grade ≥ 2 is observed, three additional courses are possible). Next cohort receives triple-hit courses (d1, d2, d3) every 4 weeks. The third application scheme includes quintuple-hit courses (d1, d2, d3, d4, d5) administered intravenously every 4 weeks.^28^ Results presented at ASCO 2013 (ref. 29) showed that GL-ONC1 applied in these regimens was well tolerated (only 1 in 33 patients experienced a dose-limiting toxicity); best response was stable disease accessed at 8–12 weeks (5/33 patients) and at > 24 weeks (6/33 patients).

In a phase 1/2 trial on patients with advanced peritoneal carcinomatosis (NCT01443260) GL-ONC1 is administered into the peritoneal cavity via catheter every 4 weeks for up to four courses (see Figure 4b). Preliminary data presented at ASCO 2013 showed that GL-ONC1 was well tolerated and a reduced number of malignant cells in ascites fluid was observed.^30^

#### Single-shot application schemes in GL-ONC1 trials.

In another phase 1 trial enrolling patients with lung cancer/malignant pleural effusion (NCT01766739), GL-ONC1 is administered as a single-shot into the pleural cavity (Figure 4c). As JX-929 and GL-ONC1 both are still situated in early phase 1/2 trials focusing mainly on tolerability and safety aspects, a lack of conclusive data regarding a comparison of application schemes and corresponding therapeutic efficacies for these two different vector systems is evident. In this context, it would be tempting to initiate head-to-head vector comparisons (employing identical routes of vector application in the same types of cancer).

### Herpes simplex virus (HSV)-based oncolytic virotherapy

**Figure 5:**
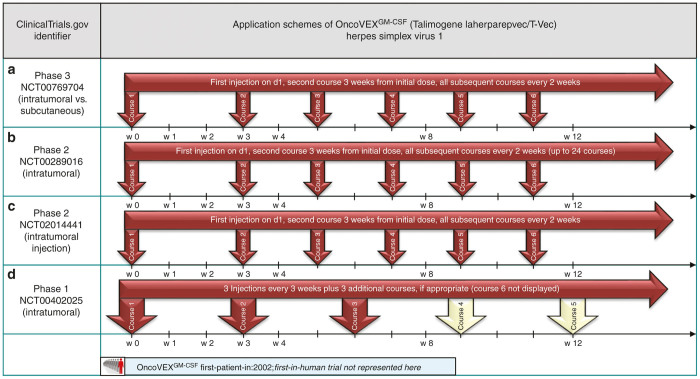
Selected application schemes for Talimogene laherparepvec (**a**–**d**): (**a**) presented at ASCO 2014,^32^ (**b**) published by Senzer *et al*.,^35^ (**d**) presented at ASCO 2012.^36^

Talimogene laherparepvec (T-Vec, OncoVEX),^31^ a HSV-1-derived herpes virus expressing GM-CSF, recently has completed a phase 3 trial on patients with advanced malignant melanoma (NCT00769704, Figure 5a). Showing a significant improvement in durable response rates compared to a GM-CSF-treated control group,^32^ thus Talimogene laherparepvec is currently (02/2015) undergoing US Food and Drug Administration^33^ and European Medicines Agency^34^ approval as a monotherapeutic agent for metastatic melanoma.

#### Repetitive application schemes employing T-Vec.

As the application scheme used in this phase 3 trial is undoubtedly of elementary interest, it is described in detail: to achieve seroconversion in seronegative patients (for safety reasons), this particular subgroup received an initial injection to reduce systemic and loco-regional adverse events. Three weeks after the initial dose, up to 10 tumor sites were injected and this procedure was repeated every 2 weeks for up to 24 times, if responses could be detected (application scheme depicted in Figure 5a).^35^ This explicit application scheme has also been used in the previous phase 2 trials (*e.g*., ref. 35 NCT00289016, Figure 5b) as well as in a trial to investigate viral biodistribution and shedding for melanoma patients (NCT02014441, Figure 5c).

In a phase 1 trial on pancreatic cancer, T-Vec was injected intratumorally every 3 weeks for up to six courses (NCT00402025, Figure 5d).^36^

Beyond that, also clinical application schemes of four other herpes-based virus platforms, which are all situated in early phase 1/2 clinical trials, were evaluated. Interestingly, also single-shot (dose-escalating) application schemes could be found for the vector systems M032 (NCT02062827; see Supplementary Figure S2C), Seprehvir/HSV 1716 (*e.g.*, NCT02031965; see Supplementary Figure S3a–c), HF10 (stage 1 of NCT01017185; see Supplementary Figure S4a) and rRp450 (NCT01071941; see Supplementary Figure S4b).

An extension of virus injection is intended to a maximum of four applications, if no severe adverse events are observed and dosing intervals vary between 1 to ≥ 2 weeks (Seprehvir, *e.g.*, presented at ASCO, 2013 (ref. 37) and HF10 presented at ASCO, 2014 (ref. 38)).

Taken together, T-Vec exhibiting the most extensive clinical evaluation so far could thus function as a prime example to substantiate the scenario of multiple application schemes, as T-Vec provided a proof-of-principle that repetitive intratumoral injections of an oncolytic virus do not only affect even noninjected tumor sites but show beneficial survival rates. Beyond current clinical data, the role of virus-mediated antitumoral immune response induced by T-Vec will be assessed in separate single-arm trial on late-stage melanoma (NCT02366195).

### Adenoviridae-monotherapy in clinical trials

The history of oncolytic virotherapy is closely linked to the development of adenovirus-derived anticancer viruses. As early pioneer clinical trials with engineered vectors, *e.g.*, the E1B-55kD-gene-mutated Adenovirus ONYX-015,^39^ provided valuable insights regarding safety aspects but revealed poor potency of those first generation oncolytics.^40^ Nevertheless, in 2006, the ONYX-015 derivative Adenovirus H101 was the first approved oncolytic vector for tumor therapy in China.^41^ Today, more therapeutically potent adenoviruses are tested as monotherapeutic agents in several trials (mostly phase 1/2).

#### Repetitive application schemes for oncolytic adenoviruses.

**Figure 6:**
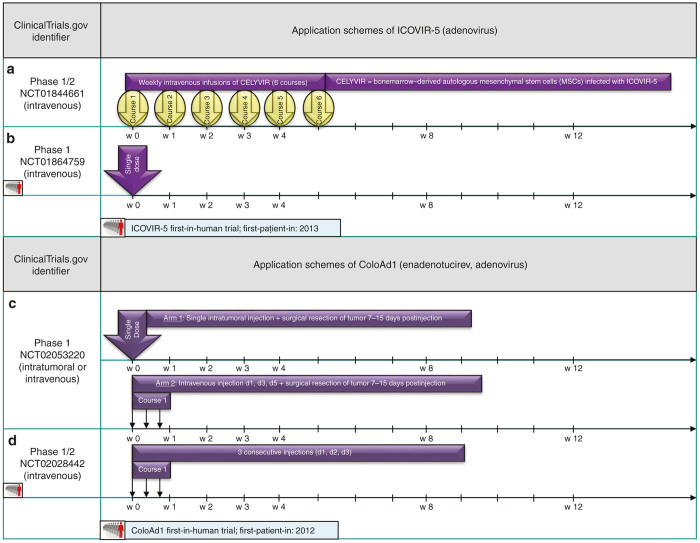
Selected application schemes for Adenoviridae ICOVIR-5 (**a+b**) and ColoAd1 (**c+d**): (**c**) presented at ASCO 2014.^47^

As an innovative approach to enhance delivery of the oncolytic adenovirus to the tumor sites, autologous mesenchymal stem cells are infected with ICOVIR-5 and administered intravenously in a phase 1/2 trial in children and adults with refractory solid tumors (NCT01844661, Figure 6a). The results of this concept have been published in the form of an exploratory study^42^ and a case report on a 9-year-old girl diagnosed with glioma.^43^

Appreciating the success of other viruses designed to amplify both cancer-cell destruction with release of tumor-specific immunogenic epitopes and subsequent immunostimulation through expression of, *e.g.*, GM-CSF,^3,44^ CG0070, an Ad5 serotype Adenovirus, which is dependent on Rb (retinoblastoma)-defectiveness, is also coding for GM-CSF. CG0070’s phase 1 trial on bladder cancer (NCT00109655) compared weekly intravesical instillations to intervals of 4 weeks with up to six courses each (see Supplementary Figure S5a). The complete response rate was for both groups together 63.6% with even 81.8% in patients with altered phosphorylation status of RB,^45^ qualifying for a subsequent phase 2/3 trial (NCT01438112, Supplementary Figure S5b): here, the study design intends for six courses of intravesical instillations in weekly intervals. The route of administration is hardly comparable to other trials as this is an elegant procedure (i) to achieve high concentrations of infectious particles at the tumor site and (ii) to spare systemic toxicities down to the minimum, which cannot be applied to most other malignancies.

#### Adenovirus: single-shot application schemes.

ColoAd1 (Enadenotucirev), an Ad3/Ad11p chimeric adenovirus,^46^ is tested in patients with colorectal cancer (NCT02053220). A single-shot intratumoral injection is compared to a triple-hit i.v. administration with subsequent tumor resection in both arms (see Figure 6c).^47^

This trial represents a systematic evaluation of Enadenotucirev delivery and spread in colorectal cancer tissues comparing the routes of systemical and intralesional administration. As a result, virus activity was detected in samples of both i.v. and intratumorally treated patients and CD8+ cell tumor infiltration was considered to be linked to an immunostimulating effect of ColoAd1.^48^ This type of analytic procedure, if complemented with data regarding efficiency, provides a solid basis for further clinical development of the most methodically sound application scheme.

The intravenous triple-hit-course application scheme (d1, d3, d5) is also part of the ColoAd1 first-in-human trial on solid tumors of epithelial origin or metastatic colorectal cancer (NCT02028442, Figure 6d).

ICOVIR-5, that employs a deregulated E2F-Rb (retinoblastoma) pathway which is very common in tumor cells,^49^ is evaluated in a single-shot intravenous dose-escalation study in melanoma patients (NCT01864759; Figure 6b).

The arsenal of oncolytic adenoviruses as monotherapy in clinical trials is completed by the so called Delta-24-RGD Adenovirus (DNX-2401), which exploits Rb/p16 deficiencies in cancer cells for replication processes.^50^ DNX-2401 entered clinical trials as a monotherapeutic particle in a phase 1 trial on brain-cancer patients, administered as single-shot therapy directly into the tumor (NCT00805376; see Supplementary Figure S5c). Results of a phase 1 trial on recurrent gynecological malignancies (NCT00562003; see Supplementary Figure S5d) were published by Kimball *et al*.^51^: Delta-24-RGD was administered into the peritoneal cavity through a catheter as a single triple-hit course (d1, d2, d3). No treatment-related severe adverse events were noted, but antitumor activity could be declared as rather modest (14 of 21 patients with stable disease as best response after 1 month).

### Applying Reovirus in cancer therapy

A recent review on reovirus-based virotherapy listed a total number of 32 clinical trials initiated since the year 2000, using a wild-type reovirus (Dearing strain) Reolysin, as monotherapeutic treatment or in combination with chemotherapy or radiation.^52^ Here, we present a selection of Reolysin monotherapy application schemes.

#### Single-shot application schemes for Reovirus.

**Figure 7:**
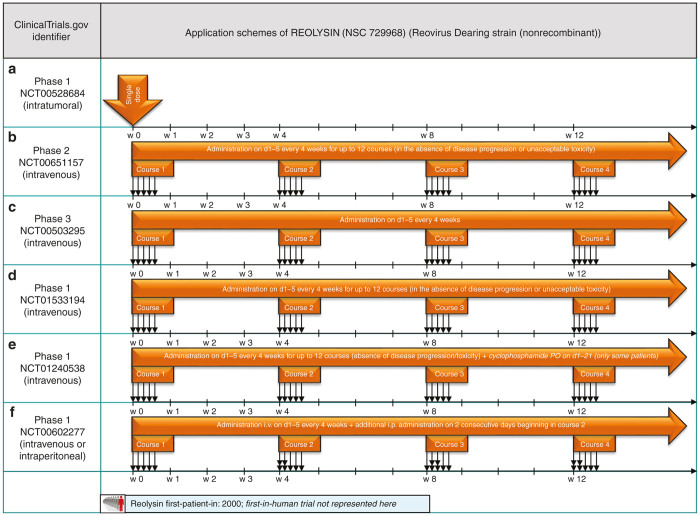
Selected application schemes for Reolysin (**a**–**f**): (**a**) published by Forsyth *et al*.,^53^ (**b**) Published by Galanis *et al*.,^24^ (**c**) presented at ASCO 2009.^54^

In a dose-escalating phase 1 trial, Reovirus was injected into lesions of recurrent malignant gliomas using the single-shot technique (NCT00528684; see Figure 7a).^53^ Reovirus treatment caused no grade III or IV adverse events and the maximum tolerated dose was not reached. Due to protocol-design, no precise information was gained about treatment efficacy.

#### Reovirus: repetitive application schemes.

The first phase 2 trial (NCT00651157) applied a different application scheme to treat patients with metastatic melanoma: the protocol demanded systemic infusion as a quintuple-hit-course (d1, d2, d3, d4, d5) repeated every 4 weeks (see Figure 7b). Galanis *et al*.^24^ reported that none of the 21 patients enrolled had an objective response and median time to progression was found to be 45 days and median time to survival 165 days. Repeated systemic administration using courses of OV application on 5 consecutive days (“hit hard and early” paradigm) so far only has been undertaken with reovirus. But, despite evidence for virus replication in tumor samples, lack of antitumor efficacy was found. Thus, a systematic comparison of alternative dosing schedules could help to identify more potent application schemes.

The same quintuple-hit-course (d1, d2, d3, d4, d5), repeated every 4 weeks, was also object of a phase 2 trial on bone and soft tissue sarcomas (NCT00503295, Figure 7c), resulting in a stable disease rate for more than 2 months for those patients in 42%.^54^ The application scheme described here seems to be well established for Reolysin as it is also standard practice in trials NCT01533194 and NCT01240538 (see Figure 7d,e).

A slight adaption to the established infection schedule is made for the NCT00602277 phase 1 trial on patients diagnosed with ovarian/peritoneal cancer: interestingly, two different routes of administration are chosen, as the i.v. quintuple-hit course (d1, d2, d3, d4, d5) is amplified by a double-hit course (d1, d2) with intraperitoneal administered Reolysin (see Figure 7f). This procedure is repeated every 4 weeks until progression of disease or appearance of unacceptable adverse events.

Notably, as both data from preclinical studies and clinical trials using monotherapeutic approaches revealed higher efficacies for Reolysin therapy in combination with radiation or chemotherapy, the main part of the currently ongoing Reolysin trials involve combinatorial modalities that were explicitly excluded in this review (see also above).

To sum up those results, we found the majority of ongoing and completed clinical trials employing oncolytic viruses for cancer treatment to be in early phase trials with a very limited number of treated patients. Systematic acquisition of tumor biopsy data including key virus parameters as well as immunological data often was not undertaken (see also discussion below).

In order to provide a comprehensive outline for the readers, application schemes for vector systems not abovementioned are displayed as supplementary figures. These include Sabin Vaccine Polio-Virus (PVS-RIPO, Supplementary Figure S2a), Parvovirus H-1 (ParvOryx, Supplementary Figure S2b), Herpes Simplex Virus 1 (M032, Supplementary Figure S2c), Coxsackievirus A21, (CAVATAK, Supplementary Figure S6a–d), Seneca Valley Virus-001 (NTX-010, Supplementary Figure S7a,b), and Vesicular Stomatitis Virus (VSV IFN-β, Supplementary Figure S7c).

## Discussion and Conclusions

Unsurprisingly, our assessment of current virotherapy application schemes came to the finding that there is yet not any “gold standard” for the design of application schemes for oncolytic viruses. In accordance with that, a huge diversity of recent clinical virotherapy trials encompassing multiple procedures of single-dose dispensations as well as a large collection of repeated application schemes is depicted (Table 1).

**Figure 8:**
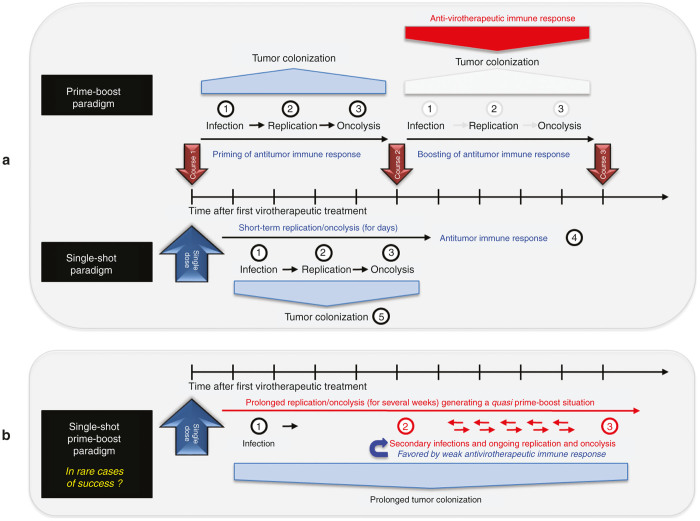
Prime-boost/single-shot paradigms of virotherapeutics application schemes: (**b**) The prime-boost paradigm (upper panel) encompasses a huge variation in the number of application courses of oncolytic viruses either applied as single-hit (d1 only) or multiple-hit courses (d1, d2, …, dx). Here, priming of an antitumor immune response (depicted in the left part of the panel) is the result of initial tumor cell infection and colonization (①), replication (②) and subsequent oncolysis (③). After eventual decrease of this primary antitumor immune response, the second and every following course of repetitive virus application is used under the premise (i) to further debulk remaining tumors using once again mechanisms of direct virus-mediated oncolysis (①+②+③) and (ii) boosting the antitumor immune response (depicted in the right part of the panel) by releasing concealed tumor antigens within the meaning of an antitumor vaccination.^3^ Preferential route of administration here is an intratumoral injection, as a rapid neutralization of viruses by a simultaneously triggered antiviral immune response (depicted by a red arrow-type rectangle) can be avoided. In addition, multiple-hit courses in the prime-boost setting are limited by an antiviral immune response as well, since the adaptive immune response is fully qualified often at the latest 7 days after the first injection and thus further virus applications are considered as ineffective. Therefore, intervals between courses have to find a balance between attacking the tumor as soon as possible and simultaneously avoiding premature neutralization of the virotherapeutic vectors. The single-shot paradigm (lower panel) is in accordance with the initial understanding of the “Oncolytic virotherapy paradigm”,^10^ as it is believed that a single systemic administration of oncolytic viruses leads to a systemic spread with subsequent selective primary infection (①) of the primary site of the tumor as well as of disseminated metastases. Self-amplification/replication (②) of virotherapeutic vectors is followed by direct tumor cell (onco-)lysis (③) and recognition of infected tumor cells by the innate host immune system with subsequent clearance of residual tumor masses through a tumor antigen triggered adaptive host-immune response (④). Basic prerequisite for a successful utilization of the single-shot paradigm is to maximize the initial dosage of applied infectious particles as dose-dependent tumor colonization (⑤) and subsequent oncolysis of disseminated tumors is only achievable if a viremic threshold is passed.^50^ Below this threshold, systemically administered virus particles are immediately neutralized by preexisting antibodies or serum factors, such as complement.^69^ (**b**) The prime-boost paradigm in rare cases of success addresses rare patient specific defects in the antiviral immune response being so far undetected and clinically silent. Thereby, a prolonged replication/oncolysis (for several weeks) generating quasi prime-boost situation is probably generated with the help of nature.

When coming to an interpretation of this current state of the virotherapy field, it first has to be pointed out that so far not any virotherapeutic vector has been approved for routine use in clinical practice. This step is still awaited, reflecting that so far no clear-cut phase 3 breakthroughs have been achieved in the field. Second, prime examples for success achieved so far in virotherapy have to be discussed and correlated with the respective application regimens which might have fostered these (rare) success stories. This kind of analytic view quite stringently leads to the conclusion that not only one, but presumably two quite divergent application strategies could lead to success, *i.e.*, “hit hard and early” and “killing softly”, reflecting also the two quite opposite paradigms of “single-shot” and “prime-boost” regimens (Figure 8).

To start with, the “single-shot” paradigm (Figure 8a, lower panel) is best represented by the recent report on two measles-seronegative patients with relapsing drug-refractory myeloma being both treated by a single-shot intravenous infusion with a very high dosage of the measles vaccine virotherapeutic MV-NIS leading in one patient to a durable complete remission at all disease sites.^17^ Key factors postulated to have contributed to this successful outcome were mentioned as follows: (i) low pretreatment serum titers of antimeasles antibodies; (ii) usage of a very high virus dosage being sufficient to overcome a postulated dose-threshold required for successful tumor colonization; (iii) detection of measles virus transcripts (but not of live virus particles) in circulating cells even at 6 weeks after virus infusion, by which time there had been a substantial boost to the antimeasles antibody titer, suggesting the possibility of a continuing oncolytic activity even at that late time (Figure 8b).

The “prime-boost” paradigm (Figure 1, upper panel) is best represented by the recent OPTiM phase 3 virotherapy study in which the HSV-1 based, GM-CSF encoding virotherapeutic vector talimogene laherparepvec (T-Vec) was applied intralesional/intratumoral in unresected stage IIIB/C and IV melanomas.^55^

Following an initial dose (functioning as a priming of the antitumor immune response; Figure 8a, upper panel, on the left), dosing of T-Vec was repeated every 2 weeks for up to 24 times defining a therapy intense “multiple-shot/long-term application” scenario, being in maximum contrast to any of the single-shot scenarios. Based on this application regime, T-Vec proved it could shrink tumors, keep them from regrowing and improve median survival. However, T-Vec hit its primary endpoint of durable response but missed its second goal of boosting overall survival (*P* value of 0.051; see: http://www.fiercebiotech.com/story/will-fda-advisers-shoot-down-amgens-cancer-vaccine/2015-02-12).

As premises for successful oncolytic virotherapy are multifactorial and multidimensional, several key factors contribute to an enhanced treatment efficacy which can be demonstrated using the example of T-Vec skin-cancer treatment: melanoma as a tumor entity seems to be highly vulnerable to oncolysis, as virotherapeutic treatment effects were also found following JX-594 vaccinia injections.^6^ Both T-Vec and JX-594 were administered in conformance with the prime-boost paradigm, supporting to further cherish this application scheme in melanoma treatment. Both, T-Vec and JX-594 encode for human GM-CSF, an immunomodulating cytokine that is evaluated for the treatment of skin cancer as a monotherapeutic agent itself.^56^ Therefore, this particular biology of vectors T-Vec and JX-594 seem to represent a qualified approach to this particular cancer biology.

Putatively, as mentioned above, such “multiple-shot/long-term application” scenarios only can be successful if the respective virotherapeutics are applied intratumoral. Otherwise, the antivirotherapeutic immune response (depicted as a red arrow-type rectangle in Figure 8a, upper panel), which often is induced as early as 7 days after the very first virotherapeutic treatment, would completely block with great efficiency any subsequent colonization of the respective tumor sites, although this would be required for a repetitive boosting of the antitumor immune response (Figure 8a, upper panel, on the right).

The only exemptions to this rule could be postulated through a transient adjuvant usage of immunosuppressive drugs (such as cyclophosphamide) being capable to bring down the antivirotherapeutic immune response for a couple of days,^29,57–59^ or alternatively a patient-specific defect in the antiviral immune response being so far undetected and clinically silent. In both situations, a repetitive colonization of the tumor tissues could be enabled also in the case of systemically applied virotherapeutics.

Beyond that, application regimens of T-Vec (and of many other virotherapeutics) are currently combined with checkpoint inhibition-mediated immunotherapy (*e.g.*, the anti-CTLA-4 antibody ipilimumab or the PD-1 checkpoint inhibitor pembrolizumab) in order to remove the brakes from the activated T-cell response against tumor antigens.^60,61^ This novel approach could be highly instrumental in improving success of the immunovirotherapeutic “prime-boost” paradigm as discussed here. Again, this novel combinatorial approach will also require a thorough optimization of application regimens which again is suggested to be worked out by biopsy-based phase 1/2 studies (see below), a procedure which unfortunately is not yet part of the clinical development of virotherapeutics. These biopsy programs should not only monitor key virus parameters, but also key parameters of immunogenic cancer cell death. In this context, features of immunogenic apoptosis, necrosis/necroptosis, pyroptosis, and autophagic cell death should be determined and quantified^62–64^ (also reviewed in refs. 65, 66). “Dying the right way” may greatly potentiate adaptive antitumor immunity and is supposed to be directly linked to the design of virotherapeutic application schemes. Beyond that the danger signal generation (triggered by damage-associated molecular patterns coinciding with the oncolysis-triggered release of tumor-associated antigens) should also be investigated and correlated with the activation status of dendritic cells and any thereby elicited adaptive antitumor immune responses. In addition, the design of virotherapeutic application schemes is supposed to be of high importance for the adjustment of the delicate balance between antivirus and antitumor responses, being exceedingly critical to the clinical success of virotherapy.^11^

When looking back, many of the current virotherapeutics have been in clinical testing for many years (first-patient-in displayed in Figures 1–7 and Supplementary Figures S2–S7). However, when critically evaluating the application schemes being tested for these virotherapeutics all over the years, there does not seem to be a great variation/“willingness to play around much”, even when looking on the early years of the respective clinical developments. This could either mean that the application schemes being repeated with great adherence in each and every subsequent clinical trial have been proven to be optimal by study data or that virotherapy study designers heavily rely on preclinical data/animal studies (in which it is much less time consuming and much less cost intensive to vary application regimes).

Hereby, one has to keep in mind, that oncolytic viruses have either naturally occurring cancer tropism or have genetically engineered tumor selectivity among with a biologically determined tropism for a subset of cell types and, importantly, species. Therefore, most preclinical studies are performed on murine xenograft models, allowing to mimic human tumor biology but lacking immunocompetence. To access immunotherapeutic aspects of oncolytic virotherapy, there is a small number of preclinical trials on immunocompetent syngeneic mouse models. Even in these model systems, a systematic evaluation of different application schemes with varying dosing intervals and routes of administration is not standard practice so far, but should be integrated into future preclinical study protocols.

Once again, measles-based virotherapy is here taken as an object lesson for this dilemma: as described above, MV-NIS showed very promising results for further employment as an antimyeloma treatment option when administered systemically as a single-shot. To our knowledge, preclinical experiments did not methodically compare the single-shot application regimen to a multiple-hit approach in a myeloma xenograft model.^13^ But nonarmed paternal MV-Edm strain experiments compared seven dosages of intratumoral injection with intravenous administration in xenograft mouse models as well, without systematic adjustments of dosing intervals.^67^ To start from the premise that MV-NIS will be used to treat a disseminated heamatological disease, investigators thus focused on intravenous administration. Nevertheless, there is a possibility that systematic evaluation of varied dosing intervals in a multiple-hit setting could reveal an even more potent application scheme in this context, especially when carried out in an immunocompetent model system to reveal immunological interactions.

Also, absence of gross toxicities in hitherto published virotherapy trials would argue for a conservative attitude concerning a systematic modification/optimization of virotherapeutic application schemes. Raising of dosage levels and shortening of dosage intervals principally could lead to severe drawbacks in developmental programs of virotherapeutics.

More likely, start-up companies or (in very rare cases) academic research institutions who represent the upfront clinical developers in the virotherapy field by far do not have the financial capacities to optimize clinical application regimes. Therefore, the design of application regimes still seems to be a big issue concerning success in virotherapy. In this context, implementation of relative inexpensive specially designed phase 1 studies, which (i) systematically vary the application time points (and possibly also dosing levels) while (ii) systematically controlling key parameters of virotherapy (*e.g.*, rates of primary infection, replication, oncolysis, as well as antiviral and antitumoral immune effects) could be highly instrumental, especially when being grounded on a repetitive sampling and subsequent analysis of those tumor samples. On this basis, a rational design of significantly improved virotherapeutic application schemes should be possible and is even more required when it comes to combination regimens (as outlined above in case of the future usage of checkpoint inhibitors). Finally, the design of head-to-head comparisons (such as using two different virotherapeutics originating from two distinct virus families being applied via identical routes in the same types of cancer) would also of great importance to dissect which virotherapeutic would be the most efficient in defined clinical situations.

## Figures and Tables

**Table 1:** Selected clinical trials using oncolytic vector systems as monotherapeutic agents

*Virus family*	*Vector*	*Route*	*Application scheme*	*References*
Adenovirus	ColoAd1 (enadenotucirev)	Intratumoral/intravenous	*Arm 1:* Single shot	NCT02053220;^47^
			*Arm 2:* Triple-hit course (d1, d3, d5)	
		Intravenous	One triple-hit course (d1, d3, d5)	NCT02028442
	ICOVIR-5	Intravenous	Weekly intravenous infusions of bone marrow–derived autologous mesenchymal stem cells infected with ICOVIR-5 (=CELYVIR)	NCT01844661
		Intravenous	Single shot	NCT01864759
	CG0070	Intravesical	Weekly intravesical administration (6 courses)	NCT01438112
		Intravesical	*Arm 1:* Weekly intravesical administration (6 courses)	NCT00109655;^45^
			*Arm 2:* Every 4 weeks (for up to 6 courses)	
	DNX-2401 (Delta-24-RGD)	Intratumoral	Single shot	NCT00805376
		Intraperitoneal	Triple-hit course (d1, d3, d5)	NCT00562003;^51^
Coxsackievirus	CAVATAK	Intratumoral	10 intratumoral injections over 18 weeks (d1, d3, d5, d8, d22, d43, d64, d86, d106 + d127)	NCT01227551;^72^
		Intratumoral	*Group 1:* Single shot	NCT00832559
			*Group 2:* Three injections (d1, d3, d5)	
			*Group 3:* Six injections (d1, d3, d5, d7, d9, d11)	
		Intratumoral	Two injections (d1, d3)	NCT00438009
		Intratumoral	Single shot	NCT00235482
Herpes simplex virus	Talimogene laherparepvec (T-Vec/OncoVEX)	Intratumoral	First injection on d1, second course 3 weeks from initial dose, all subsequent courses every 2 weeks	NCT02014441
		Intratumoral	First injection on d1, second course 3 weeks from initial dose, all subsequent courses every 2 weeks	NCT00769704;^32^
		Intratumoral	See above; up to 24 courses	NCT00289016;^35^
		Intratumoral	Three injections every 3 weeks (plus max. three additional courses)	NCT00402025;^36^
	M032	Intra-/peritumoral	Single shot	NCT02062827
	Seprehvir (HSV 1716)	Intravenous/intratumoral	*Part 1:* Single shot; *Part 2:* plus max. three additional courses	NCT00931931;^37^
		Intrapleural	*Part A:* Single shot	NCT01721018
			*Part B: Group 1:* 2 courses at weekly intervals *Group 2:* 4 courses at weekly intervals	
		Intra-/peritumoral	Single shot	NCT02031965
	HF10	Intratumoral	*Stage 1:* Single shot	NCT01017185;^38^
			*Stage 2:* 4 courses (dosing interval ≥ 2 weeks)	
	rRp450	Into hepatic artery	4 courses every 1–2 weeks	NCT01071941
Measles vaccine virus (Edmonston strain)	MV-CEA	Intratumoral/into resection bed	*Arm 1:* Single shot	NCT00390299
			*Arm 2:* Two-hit-course (d1, d5)	
		Intraperitoneal	Every 4 weeks for up to 6 courses	NCT00408590;^14^
	MV-NIS	Intrapleural	Every 4 weeks for up to 6 courses	NCT01503177
		Intraperitoneal	Every 4 weeks for up to 6 courses	NCT00408590;^15^
		Intraperitoneal	*Course 1:* Only MV-NIS i.p.;	NCT02068794
			*Subsequent courses:* MV-NIS infected mesenchymal stem cells i.p. (every 4 weeks for up to six courses)	
		Intratumoral	Single shot	NCT01846091
		Intravenous	*Arm 1:* Single shot	NCT00450814;^17^
			*Arm 2:* Single shot in combination with cyclophosphamide	
Parvovirus	ParvOryx	Intratumoral/intravenous	Two courses (d1, d10)	NCT01301430;^71^
Polio virus (Sabin strain)	PVS-RIPO	Intratumoral	Single shot	NCT01491893;^70^
Reovirus (Dearing strain)	Reolysin	Intratumoral	Single shot	NCT00528684;^53^
		Intravenous	Up to 12 quintuple-hit courses (d1–5) every 4 weeks	NCT00651157;^24^
		Intravenous	Quintuple-hit courses (d1–5) every 4 weeks	NCT00503295;^54^
		Intravenous/intraperitoneal	Administration i.v. as quintuple-hit courses (d1–5) every 4 weeks + additional i.p. administration on 2 consecutive days beginning with course 2	NCT00602277
		Intravenous	Up to 12 quintuple-hit courses (d1–5) every 4 weeks	NCT01533194
		Intravenous	Up to 12 quintuple-hit-courses (d1–5) every 4 weeks	NCT01240538
Senecca Valley virus	NTX-010	Intravenous	Single shot	NCT01017601;^74^
		Intravenous	Single shot	NCT00314925;^75^
Vaccinia virus (Lister strain)	GL-ONC1 (GLV-1h68)	Intraperitoneal	Every 4 weeks (4 courses)	NCT01443260;^30^
		Intrapleural	Single shot	NCT01766739
		Intravenous	*Arm 1*: Every 4 weeks (up to 6 courses)	NCT00794131;^29^
			*Arm 2:* Every 4 weeks (3 triple-hit-courses d1, d2, d3)	
			*Arm 3:* Every 4 weeks (3 quintuple-hit-courses d1, d2, d3, d4, d5)	
Vaccinia virus (Western Reserve strain)	vvDD-CDSR (JX-929)	Intratumoral/intravenous	Single shot	NCT00574977;^26^
Vaccinia virus (Wyeth strain)	JX-594 (pexastimogene devacirepvec, Pexa-Vec)	Intratumoral	Three courses every 2 weeks	NCT00554372;^68^
		Intratumoral	Every 3 weeks (max. 8 courses)	NCT00629759;^21^
		Intratumoral	Weekly (up to 6 courses)	NCT00429312;^7^
		Intravenous	Single shot	NCT00625456;^23^
		Intravenous	Every 2 weeks (up to 4 courses)	NCT01380600;^22^
		Intravenous	Treatment on d1, d8, d22 and weeks 6, 12, 18	NCT01387555;^20^
		Intravenous	Weekly for 5 weeks (followed by up to 3 additional infusion boosts)	NCT01394939
		Intravenous	Weekly for 5 weeks, then every 3 weeks	NCT02017678
		Intravenous	Every 2 weeks	NCT01469611
		Intravenous	Weekly for 5 weeks (treatment extension: i.v. infusion every 3 weeks in case of stable disease)	NCT01636284
Vesicular stomatitis virus	VSV-IFN-β	Intratumoral	Single shot	NCT01628640
