# Supplementary material for: Assessment of current virotherapeutic application schemes: “hit hard and early” versus “killing softly”?
Source: Mol Ther Oncolytics. 2015 Nov 4;2:15018–. doi: 10.1038/mto.2015.18 (PMC4782955; doi:10.1038/mto.2015.18)
Supplement: Supplementary Figures [file mto201518-s1.doc]

#
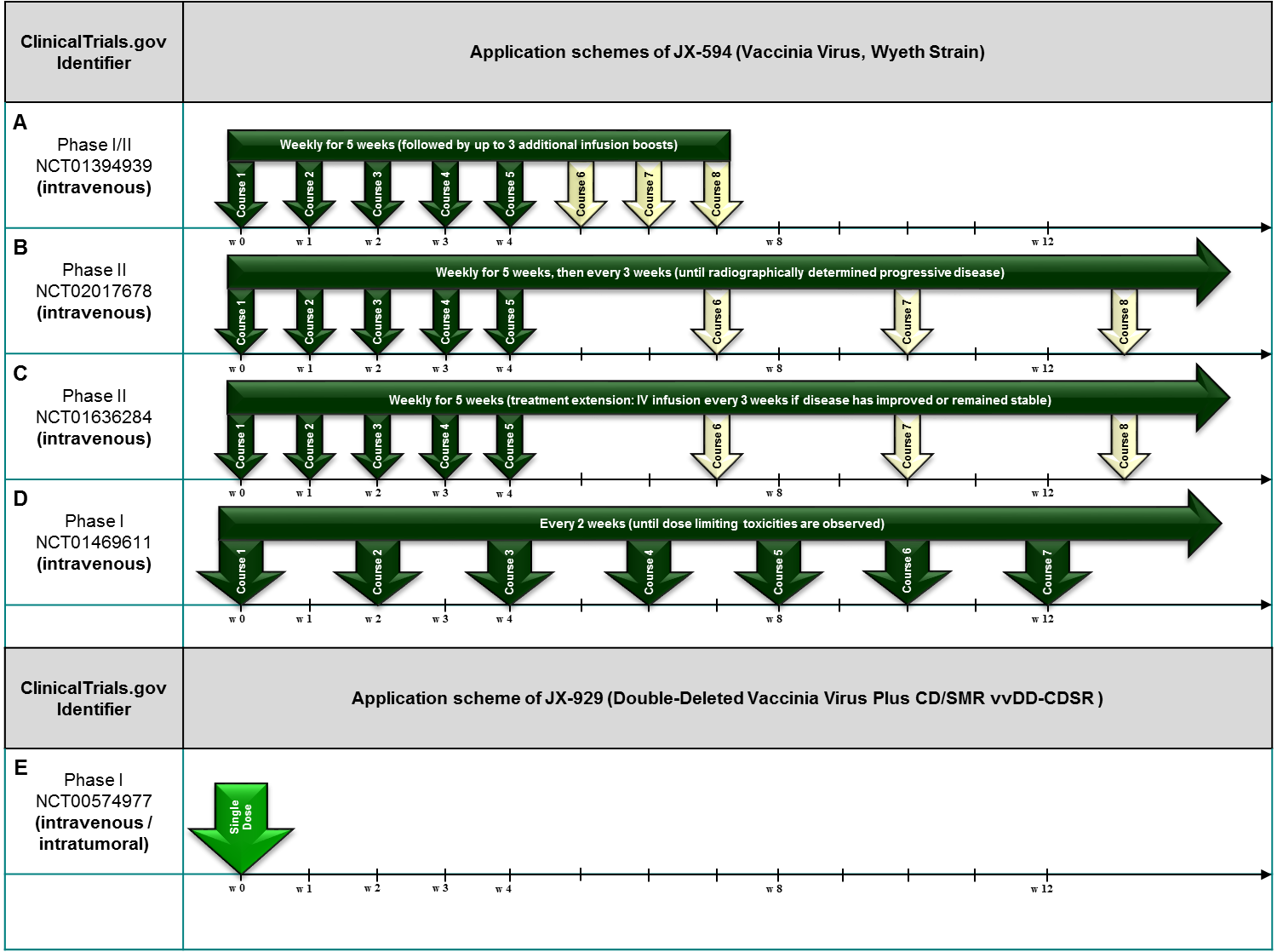
Supplementary Figures

**Supplementary Figure 1: Selected application schemes for JX-594 (A-D) and JX-929 (E):** published by Zeh et al. [26]

**
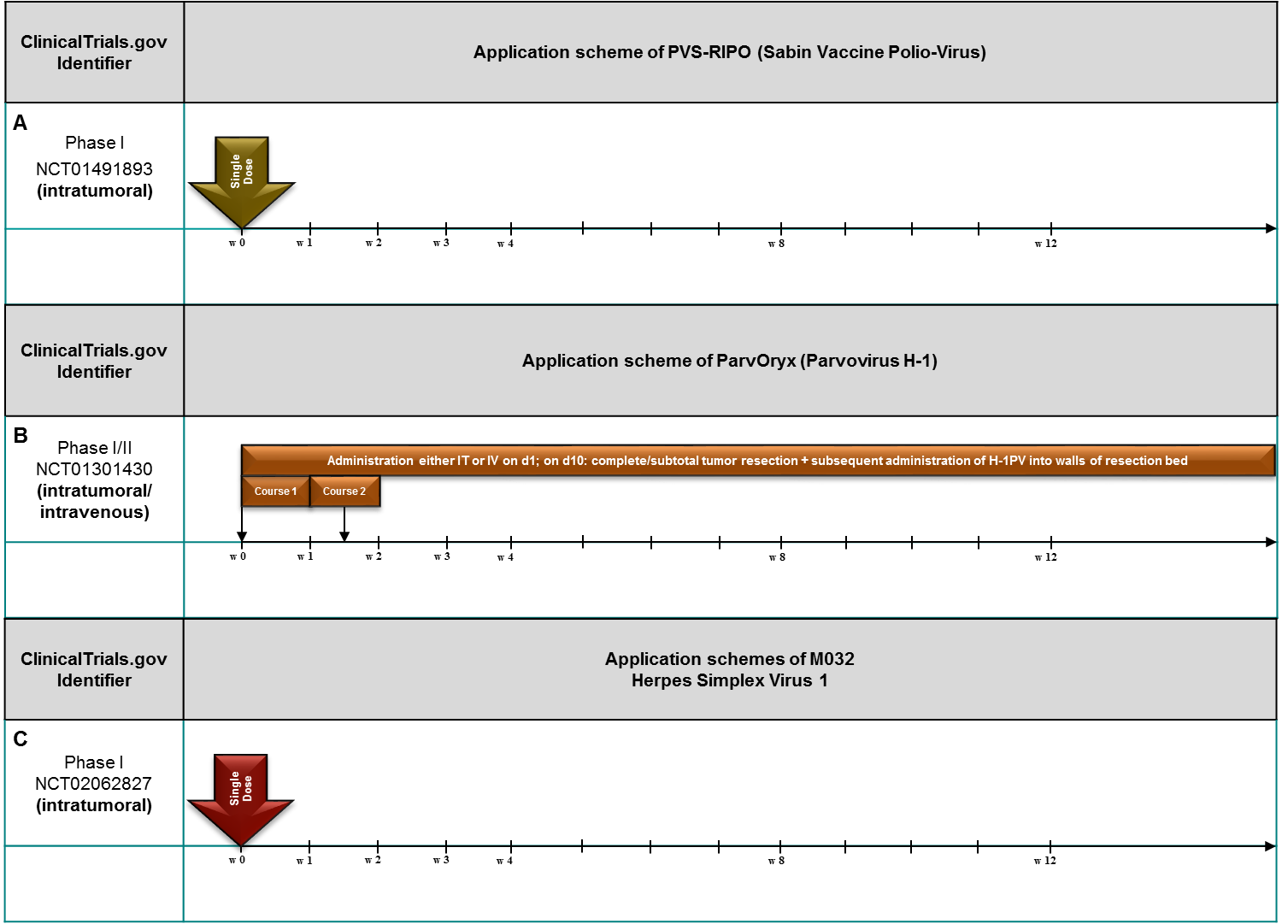
**

**Supplementary Figure 2: Application schemes for (A) polio virus PVS-RIPO (B) parvovirus ParvOryx and (C) herpes simplex virus M032: (A)** presented at ASCO 2014 [70], **(B)** published by Geletneky et al. [71]

**
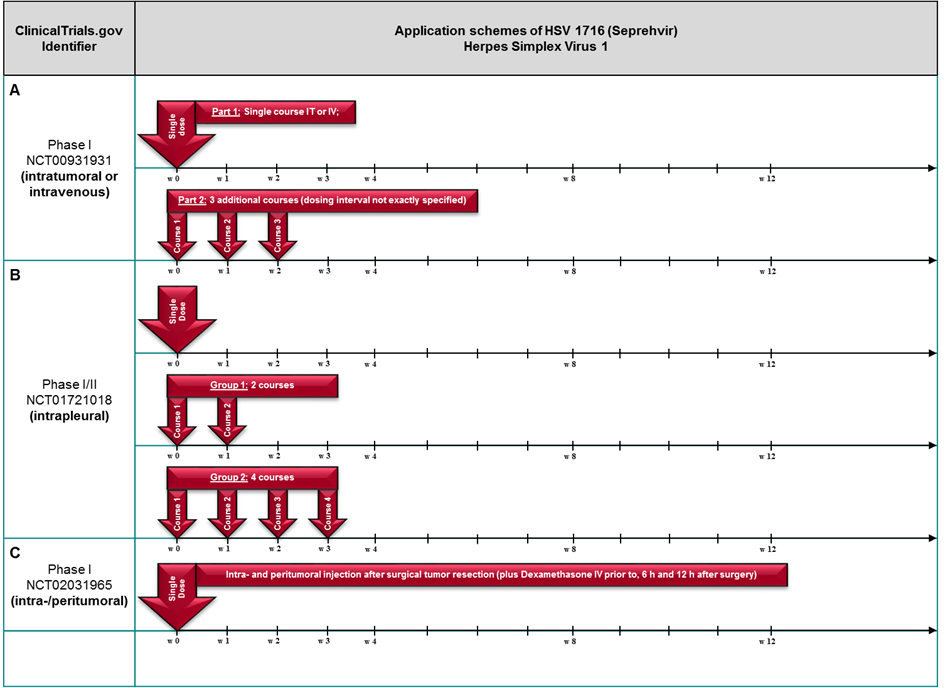
**

**Supplementary Figure 3: Application schemes for herpes simplex virus Seprehvir: (A)** presented at ASCO 2013 [37]


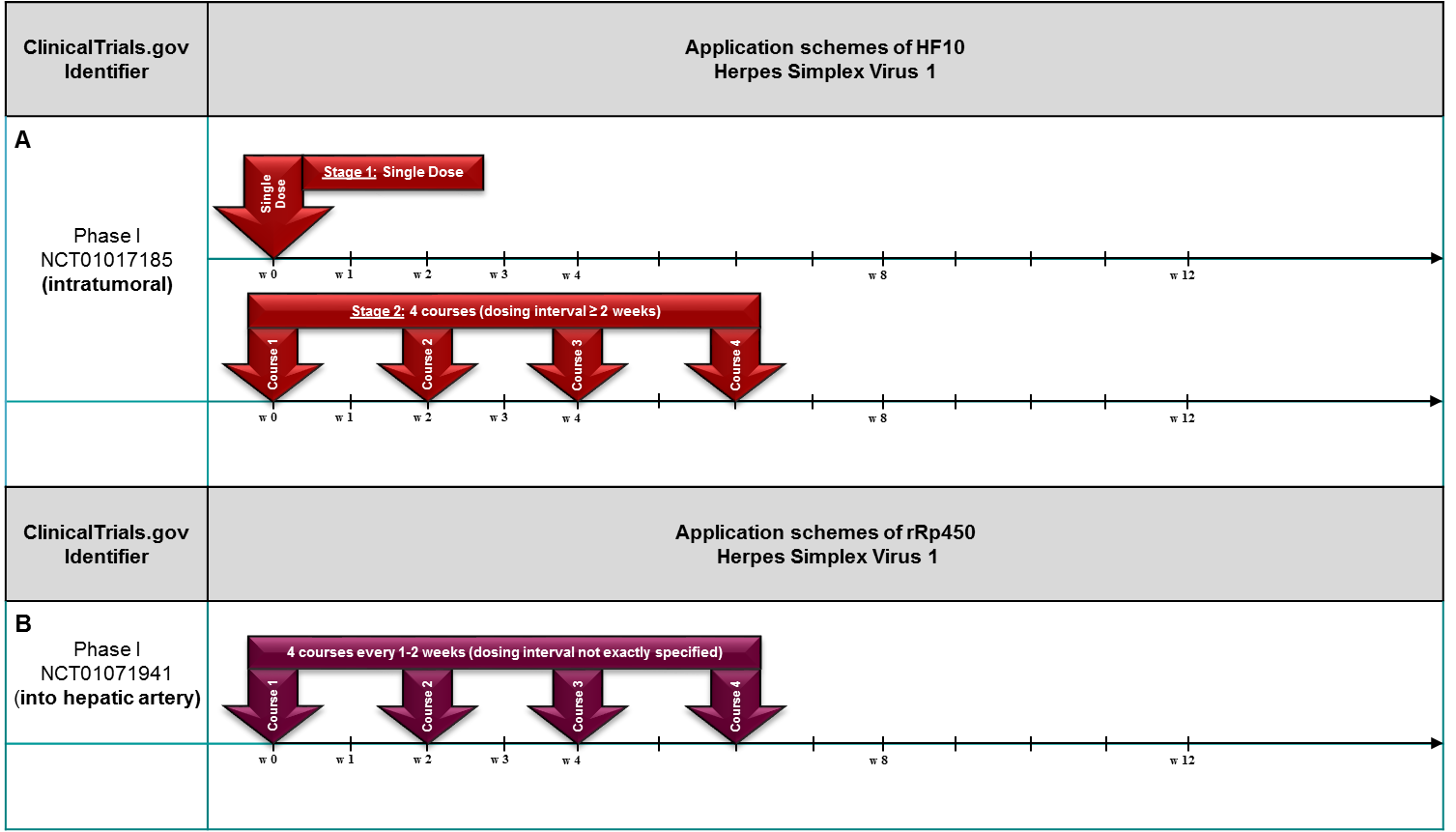


**Supplementary Figure 4: Application schemes for herpes simplex viruses (A) HF10** presented at ASCO 2014 [38] **and (B) rRp450**

**Supplementary Figure 5: Application schemes for Adenoviridae (A+B) CG0070 and (B+C) Delta-24-RGD: (A)**
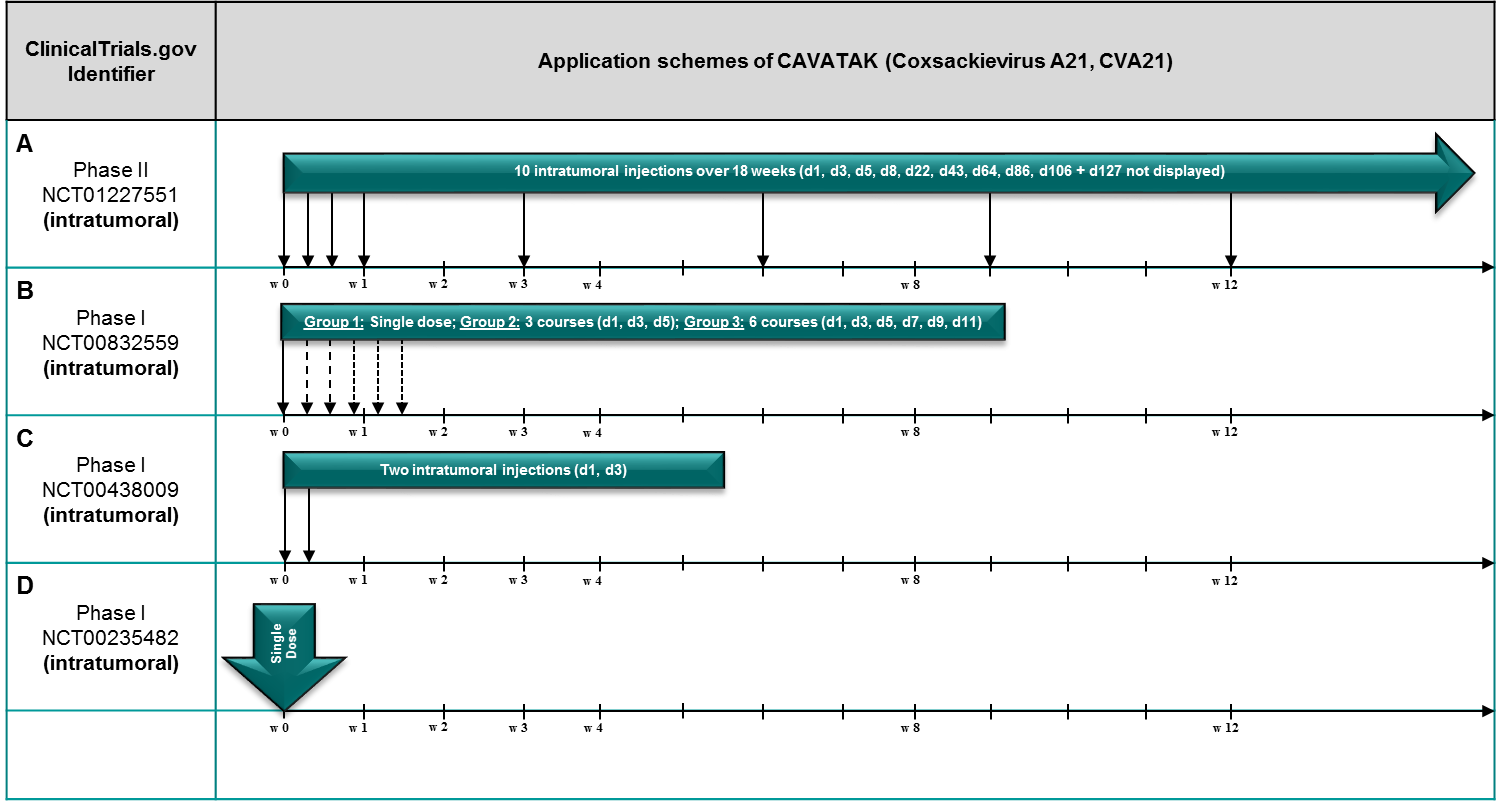
published by Burke et al.[45], **(D)** published by Kimball et al.[51]
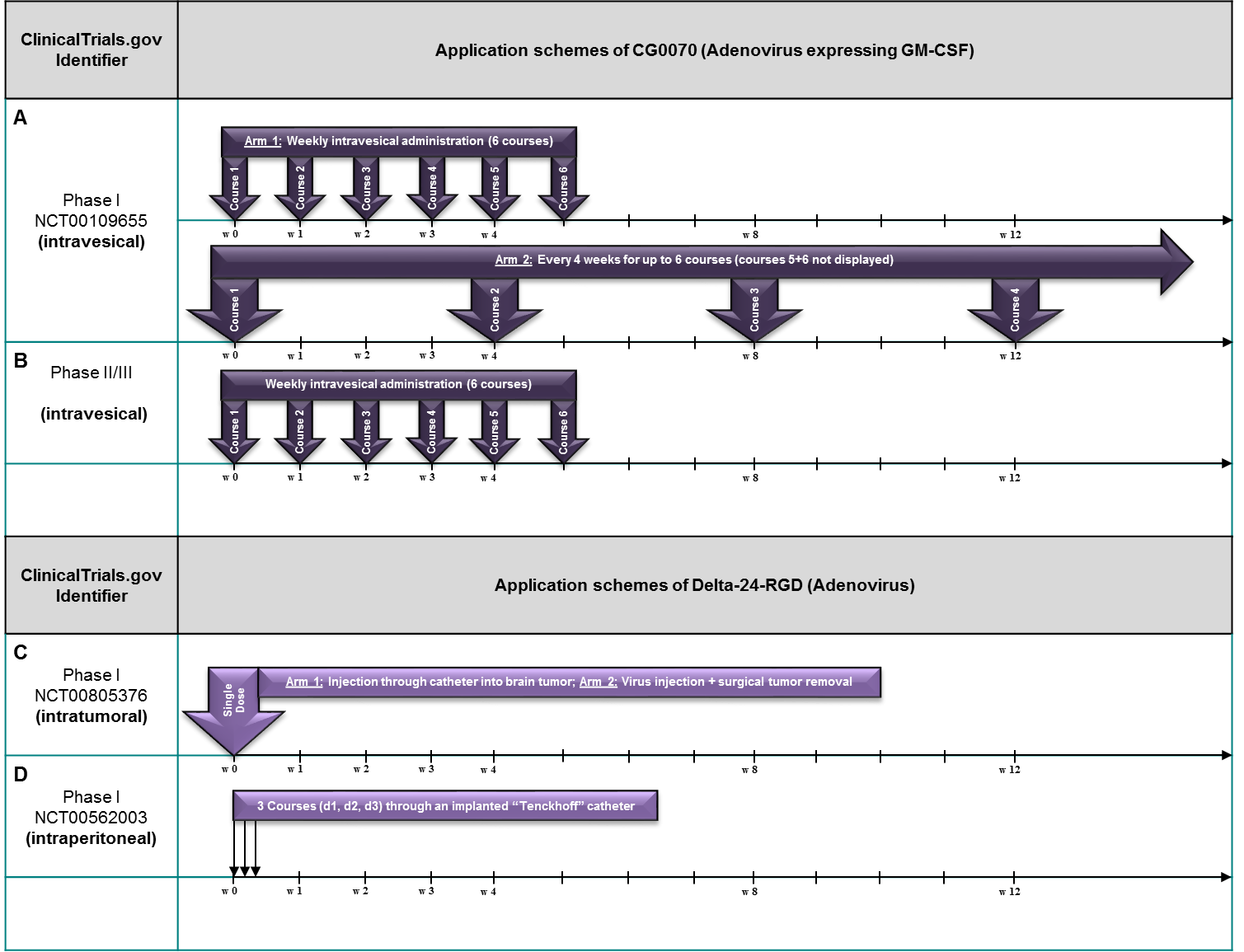


**Supplementary Figure 6: Application schemes of Coxsackievirus CAVATAK: (A)**presented at ASCO 2014 [72] and **(C)** presented at ASCO 2011 [73].


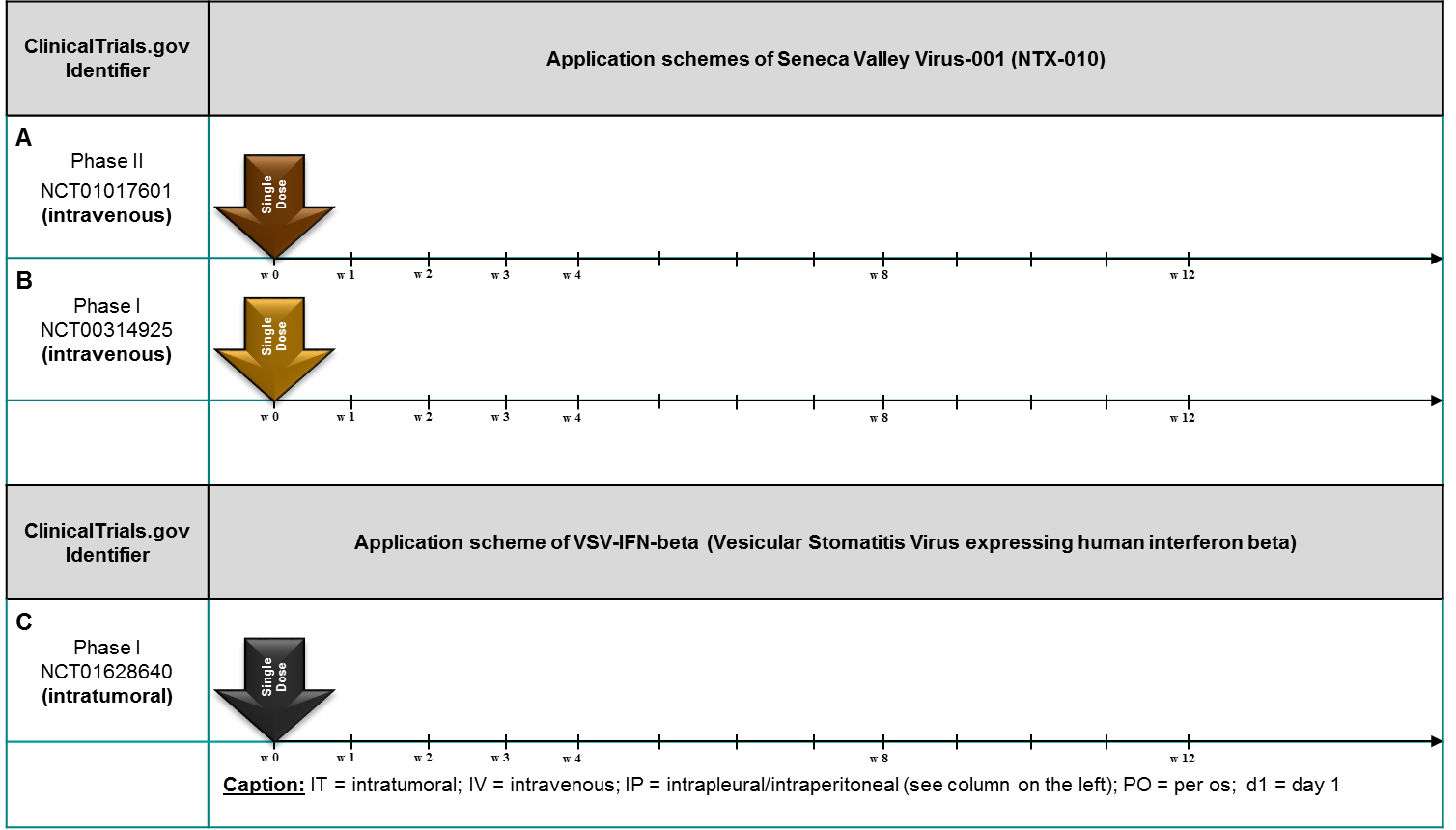
**Supplementary Figure 7: Application schemes of (A+B) Seneca Valley virus NTX-010 and (C) Vesicular Stomatitis Virus VSV-IFN-beta: (A)** presented at ASCO 2013 [74] and **(B)** presented at ASCO 2009 [75]
